# Problematic gambling in municipal social work in Tampere, Finland: Social workers’ perceptions of service pathways before the casino opening and the health and social services reform

**DOI:** 10.1177/14550725251325032

**Published:** 2025-03-19

**Authors:** Paula Jääskeläinen, Katja Kuusisto

**Affiliations:** Faculty of Social Sciences, 3835 University of Helsinki, Helsinki, Finland; Faculty of Social Sciences, 7840 Tampere University, Tampere, Finland

**Keywords:** Focus groups, gambling-related harm, municipal social workers, problem gambling, shame, stigma

## Abstract

**Aims:** Gambling can lead to a variety of economic and social harms, many of which are of central concern to social work. However, the “invisibility” of gambling-related harms can impede professional assistance by delaying recognition in social and healthcare services. The present study illuminates how problematic aspects of gambling surface in social work encounters within municipal social services, and how municipal social workers of the City of Tampere, Finland, perceive the available service pathways for problem gambling help provision before the opening of a casino in the city in 2021 and before the health and social services reform of 2023. **Methods:** We conducted five focus group interviews with 15 social workers employed by the City of Tampere Social Services, utilizing the Reception Analytical Group Interview (RAGI) method. The data were analyzed thematically using ATLAS.ti. **Results:** Gambling-related harm is entangled with multiple other issues, often inadvertently revealed in daily social work encounters. Lack of time to adequately address clients’ gambling issues and the absence of suitable services in the municipality were perceived as major structural obstacles to adequate help provision. The study participants recognized gambling-related shame, stigma and misconceptions as barriers to clients’ help-seeking. However, they did not view the opening of the casino as likely to increase harms locally. **Conclusions:** The study highlights the importance of diverse approaches in providing appropriate services for individuals facing gambling-related harm. Furthermore, it underscores the necessity of addressing gambling issues in daily social work encounters and ensuring diverse, accessible service provision.

## Introduction

While gambling is recognized as a significant public health concern, with documented social, economic and societal costs (Browne et al., 2017; Hofmarcher et al., 2020), it is a highly normalized activity worldwide (Gavriel-Fried et al., 2023; Orford, 2019). Harms associated with gambling typically include financial hardship, mental health issues, family distress, emotional burden and crime, particularly affecting marginalized and disadvantaged groups (Abbott, 2020). The present study explores how gambling-related harms were intertwined with social work practices in the municipal social services in Tampere, Finland, prior to the opening of the country's second casino in the city and before the Finnish health and social services reform in 2023.

Documenting and understanding service coverage is particularly important when gambling availability increases significantly in jurisdictions. Current research indicates that increased access, often associated with the establishment of casinos, correlates with increased gambling harms (Conway, 2015; Markham et al., 2023; Momper, 2010; O'Gilvie, 2022).

Despite variations in service provision for gambling-related harms across different countries, these services typically encompass healthcare and social care (Marionneau & Järvinen-Tassopoulos, 2022). Regardless of the social services system, gambling-related harms are undoubtedly challenges that social work professionals frequently encounter in their work due the field's strong emphasis on helping individuals with financial and social difficulties (International Federation of Social Workers, 2024). However, professionals may often be unaware that these issues are caused by gambling. Consequently, a lack of knowledge on problem gambling and gambling-related harms might hinder social workers from recognizing the problem and referring clients to adequate help (Bramley et al., 2019; Manthorpe et al., 2018; Nower et al., 2023; Rogers, 2013).

Previous Finnish studies that have addressed gambling and social services have primarily focused on social workers’ perceptions and knowledge of problem gambling and their expertise in addressing it (Egerer, 2013; Egerer & Alanko, 2015; Heiskanen & Egerer, 2019; Järvinen-Tassopoulos & Kesänen, 2020). However, these studies have not specifically examined how social workers perceive daily encounters with gambling-related harm and the service pathways for further help provision. To address this gap, before the opening of a new local casino, we conducted five focus group interviews with 15 social workers, employed by the City of Tampere Social Services. At the time of our interviews, conducted in 2020–2021, the municipality was legally obliged to provide healthcare and social services for its residents, while it simultaneously anticipated hosting the casino in partly city-owned premises. We begin with a brief introduction to potential barriers to providing and receiving help for gambling-related problems. Subsequently, we provide context for the study's setup and methodology. We then present our findings and discuss the implications of our research.

## Stigma, self-stigmatization and the invisibility of problem gambling

Problem gambling, whether considered a non-substance-related addictive disorder, behavioral dependence or societal concern, is accompanied by significant public stigma and self-stigmatization (Brown & Russell, 2020; Dąbrowska & Wieczorek, 2020; Hing & Russell, 2017; Quigley, 2022). For example, “gambling disorder” ranks among the most stigmatized mental health issues (Quigley, 2022). Similarly, problem gambling is sometimes perceived as a “self-inflicted” problem, further exacerbating stigma by portraying individuals facing it as having character flaws (Hing et al., 2016; Marko et al., 2022). Intense public stigmatization can easily lead to individuals internalizing self-stigma, potentially discouraging them from seeking help for gambling-related problems (Brown & Russell, 2020; Dąbrowska & Wieczorek, 2020; Hing & Russell, 2017; Quigley, 2022).

Moreover, financial harm caused by gambling not only induces shame in individuals, but also acts as a catalyst and intensifier of financial difficulties. Due to financial shame, individuals might even withdraw from social encounters (Gladstone et al., 2021). Shame and secrecy, along with attempts to recover financially by gambling, are among the strongest predictors for delaying help-seeking (de Ridder & Deighton, 2022; Tavares et al., 2002).

Furthermore, problem gambling not only affects individuals who gamble, but also extends its impact to their family members and close ones. Those affected experience emotional, relationship and financial harms, along with feelings of shame, to an extent that may hinder them from seeking help (Hing et al., 2013; Holdsworth et al., 2013). Alarmingly, financial abuse, intimate-partner violence and problem gambling are often intertwined (Dowling et al., 2016; Hing et al., 2022; Suomi et al., 2013). Studies have shown a significant association between problem gambling and intimate partner violence, involving the gambling individual both perpetrating violence and being victimized (Dowling et al., 2016).

Despite the serious harm caused by gambling, the “invisibility” of problem gambling itself can impede professional help. Problem gambling is typically less apparent than alcohol or substance use, leading to delayed recognition by social and healthcare professionals, as well as by those close to the individual, for an extended period (Holdsworth et al., 2013; Manthorpe et al., 2018). This invisibility allows the problem to remain concealed and worsen over time (Brown & Russell, 2020). The severity of stigmatization and secrecy is sadly evident in gambling-related suicides and suicidality, with shame and indebtedness being the leading causes (Marionneau & Nikkinen, 2022).

Moreover, problem gambling is often linked to other life challenges. For example, research indicates a common comorbidity with substance use and behavioral dependencies (Castrén et al., 2013; Cowlishaw et al., 2014). To capture the complexity of gambling-related harms, Langham et al. (2015) propose a comprehensive harm framework that considers a range of harms from tangible financial difficulties and relationship issues to crime and more subtle cultural harms, affecting individuals, families, communities and societies across both the life course and intergenerational levels. The conceptual framework is provided in Langham et al. (2015).

Previous studies have shown that Finnish social workers feel a sense of responsibility to help individuals facing gambling problems (Egerer, 2013; Egerer & Alanko, 2015; Heiskanen & Egerer, 2019; Järvinen-Tassopoulos & Kesänen, 2020). Yet, gambling and gambling-related harm are not consistently addressed in social work practice in Finland. Järvinen-Tassopoulos and Kesänen (2020) found that the issue was sometimes overlooked because gambling was not naturally part of the conversation (or simply did not arise) during a social work appointment. Furthermore, social workers occasionally expressed insecurity about their knowledge of problem gambling and hesitated to broach the topic. According to social workers, clients sometimes refused to discuss gambling altogether due to the stigma and shame associated with it (Järvinen-Tassopoulos & Kesänen, 2020). Additionally, Heiskanen and Egerer (2019) noted that Finnish social services directors strongly distinguished therapeutic treatment from the social and financial support provided by social services. In this framework, problem gambling would become more of a medical issue.

Not addressing problem gambling consistently in daily social work contrasts with the everydayness of gambling activities in Finland: Finnish people generally consider gambling to be socially acceptable and culturally embedded as part of Finnish life (Matilainen, 2017). Gambling opportunities are widespread, with electronic gambling machines (EGMs), scratch cards, lotteries and sports betting available in everyday locations, including grocery stores, kiosks and gas stations across the country. In 2019, over 78% of the population had engaged in gambling, mostly online and in everyday convenience locations. Casino gambling, in turn, was a less common activity, with only 2% of Finns visiting casinos (Salonen et al., 2020).

### Context

In 2016, the Finnish state-owned gambling monopoly, Veikkaus, organized a nationwide competition for hosting Finland's second casino. Over 30 municipalities, including the City of Tampere, submitted their applications. Although there is strong evidence on harm caused by casinos (Conway, 2015; Markham et al., 2023; Momper, 2010; O'Gilvie, 2022), the Tampere City Council did not discuss or assess the potential negative impacts. Tampere, located inland in southwestern Finland, could be considered an unusual location for a new casino because urban casinos are typically established near the country's borders to attract foreign tourists. Instead, Veikkaus expected the customer base of the Tampere casino to consist mainly of local and domestic visitors, which implies that gambling-related harm would predominantly affect locals (Eadington, 1999).

Until 2023, Finnish municipalities were responsible for organizing health and social services for their residents (Social Welfare Act, 1301/2014). However, in 2023, these responsibilities were transferred to larger wellbeing services counties, each comprising several municipalities (The Act on Organizing Healthcare and Social Welfare Services, 612/2021). Health and social services in Tampere, Finland's third-largest city with a population of around 260,000 are now organized through the Wellbeing Services County of Pirkanmaa, which serves approximately 545,000 residents. Social services professionals formerly employed by the City of Tampere are now part of the personnel of the Wellbeing Services County of Pirkanmaa.

Before the legislative reform, it was common for municipal health and social services to be organized in close cooperation with non-governmental or private organizations, as was the case in the City of Tampere during the study. Today, inpatient substance use treatment, including “addiction treatment”, is organized in a similar manner despite this organizational change, and is regulated by both the Health Care Act (1326/2010) and the Social Welfare Act (1301/2014). Furthermore, the Social Welfare Act (1301/2014) stipulates that wellbeing services counties must provide specific social work and services for individuals with substance use and other addiction issues. Many services for problematic gambling, such as the national helpline, online therapy or peer support groups, are primarily provided by third-sector organizations (Finnish Institute for Health and Welfare, 2024). By contrast, problem gambling services in the neighboring country of Sweden are explicitly mandated by legislation, making gambling-specific outpatient treatment widely available in municipal social services (Månsson et al., 2022).

At the time of our interviews, the City of Tampere did not offer specialized services for individuals facing gambling problems. Low-threshold mental health and substance use treatment services were organized by the City of Tampere Healthcare Services, whereas financial and debt counceling by social services and state legal aid offices. Other services for problem gambling relied on third-sector organizations. Furthermore, in 2017, basic social assistance was transferred from municipal social services to the national social security system (National Social Insurance Institution, Kela), leaving many individuals without contact with municipal social services.

To date, research has not examined municipal social workers’ perceptions of their daily encounters with gambling-related harm or the service pathways for addressing problem gambling. To fill this gap, our study aims to explore the following questions prior to the expansion of local gambling opportunities:

1. How do the problematic aspects of gambling intertwine with social work practices?
2. How did the municipal social workers perceive the available service pathways as contributing to the help provision for gambling problems before the casino and the Finnish health and social services reform?

## Methods

The data consist of five online focus group interviews involving 15 social workers (14 women and one man) employed by the City of Tampere Social Services. The interviews were conducted between December 2020 and June 2021, just before the opening of a new casino in Tampere. Due to the ongoing Covid-19 restrictions in Finland, the interviews were held via Zoom (https://www.zoom.com) instead of in person. Our initial plan was to form groups of social workers working in different teams within the city's social services. However, due to challenges in recruiting during the pandemic, we contacted the City of Tampere Social Services directors (*N* = 22) who emailed our invitation to their teams. Potential participants were encouraged to join in focus group interviews and discuss their thoughts on the forthcoming Tampere casino, gambling-related harm and how these issues are apparent in the daily work. We emphasized that the participants were not required to have special expertise on casinos or gambling.

Out of the city's 22 social services teams, five were ultimately able to participate. The groups consisted of social workers from the same team due to challenges in scheduling, with group sizes ranging from two to four participants. Three of the groups represented the city's social services for adults, one for elderly people and one for young people (Table 1). Over the past decade, the National Institute for Health and Welfare (THL) has provided voluntary training for social work professionals on addressing gambling-related issues. Each group appeared aware of these training opportunities, and a few participants mentioned having attended them. Furthermore, four of the participants mentioned that they had previously worked in treatment services for mental health and substance use issues, which may have given them more knowledge about problem gambling.

**Table 1.** The interviewed groups.

Focus group		Duration of the discussion
1	Social services for adults	50 min
2	Social services for elderly	65 min
3	Social services for young people	90 min
4	Social services for adults	90 min
5	Social services for adults	70 min
Total	Fifteen participants (14 female, one male)	365 min

While our data are not a representative sample of the city's social workers, the data are saturated in terms of the number of groups and topics covered, given the “information power” derived from discussions with professionals from a specific city and recurring content in the data. This new knowledge builds on earlier findings regarding Finnish social workers’ perceptions of gambling, suggesting that the number of interviews conducted was sufficient (Hennink & Kaiser, 2022; Malterud et al., 2016).

All participants gave their informed consent to participate in the study. They were informed that they could refrain from answering any questions or withdraw their participation without facing any negative consequences. The interviews were conducted through an encrypted Zoom link, and both video and audio were recorded. The duration of the interviews ranged from 50 min to 90 min. Subsequently, the interviews were transcribed verbatim and anonymized.

Focus group interviews with social workers are typically utilized to gather information about participants’ perceptions of or attitudes on a specific topic (Linhorst, 2002), particularly when dealing with distant or abstract topics (Liamputtong, 2016). To stimulate free discussions drawing on participants existing knowledge and perceptions, we employed the Reception Analytical Group Interview (RAGI) method, incorporating visual film clips within focus group settings (Sulkunen & Egerer, 2009). Given the ubiquity of gambling opportunities in Finnish everyday settings, we provided the focus groups with stimuli ranging from the rare activity of casino gambling to the common activity of EGM gambling in supermarkets. This approach aimed at encouraging participants to reflect on the cultural contexts and freely associate these with their work practices.

In the first part of the focus group discussions, we presented two movie clips related to casino gambling and a marketing video featuring the upcoming Tampere casino, produced by the City of Tampere. Each vignette was followed by a series of questions designed to facilitate discussion. The interview protocol is presented in Table 2. The first movie scene was extracted from the American film *The Cooler* (Kramer, 2003), depicting gambling in a Las Vegas casino, comprising a setting likely familiar to participants from popular culture movies. The second movie scene was from *Drifting Clouds* (Kaurismäki, 1996), a film shot at the then only casino in Finland, representing a rare gambling environment in Finnish culture. Participants received no information about the movies or their plots; the purpose of displaying two different casino environments was to stimulate discussion.

**Table 2.** The interview protocol.

Part 1. Construing images of casino gambling		
	The story	Interview questions
Scene from the movie *The Cooler* (Kramer, 2003), lasting 55 s	A man enters a crowded casino. As he walks around, he touches a roulette table. The croupier smiles at him. Next, the camera turns to frustrated and angry people losing money at the game tables and EGMs and then moves to people queuing at an ATM for cash.	(1) Describe what happens in the movie scene. What are the characters in it like? (2) What kinds of thoughts does this scene evoke? (3) Could this happen in real life?
Scene from the movie *Drifting Clouds* (Kaurismäki, 1996), lasting 75 s	A couple goes to the casino. The woman sits down in the lobby and hands an envelope to the man. He enters the casino and heads to the roulette table. While waiting, the woman falls asleep. When the man finally returns, he wakes her up and says, “I lost. Everything”.	(1) Describe what happens in the movie scene. What are the characters in it like? (2) What kinds of thoughts does this scene evoke? (3) What will the situation of the characters be after a few years? (4) Could this happen in real life?
**Part 2. Construing images of Tampere as a casino location**		
Marketing video (City of Tampere, 2016), lasting 64 s	A marketing video on YouTube introducing seven possible real-estate locations for the casino in the city. The video shows architectural illustrations of these locations being set up as casinos with other typical casino elements, along with uplifting music.	(1) What kinds of thoughts does the video evoke? (2) What kind of a city does Tampere appear in the video? Does the casino fit in Tampere? (3) What are the pros and cons of opening a casino? (4) What kinds of implications could the Tampere casino have on your work or social work in general?
**Part 3. Construing gambling-related harm in Finland**		
“EGM gambling in supermarkets” (read by the interviewer)	66-year-old Aino has been an occasional EGM gambler in supermarkets and gas stations for decades. When her gambling has been more active, her husband has also remarked on it. During the recession of the 90 s, when their children were small, the family received financial help from the social services. Aino herself does not recall whether her gambling had anything to do with the situation. Since then, financial support has not been needed, although according to Aino, money has always been short. Aino retired a few years ago. Fortunately, their living costs are quite reasonable, and her retired husband receives a moderate pension too. However, Aino still does not seem to be able to get by on her pension. Therefore, gambling winnings bring a nice boost to the financial situation. The grandchildren, whom Aino likes to take care of, also bring joy to her life. One day, Aino gets a call from her granddaughter's daycare after closing time. Her granddaughter has still not been picked up. “Is it a schedule confusion? Shouldn’t Grandma have picked her up?”	(1) How and why did Aino end up in this situation? What is the cause of the situation? (2) What happens next? (3) Does Aino need help in this situation? How could you help Aino in social work?
“Casino gambling” (read by the interviewer)	34-year-old Mikko started gambling casino table games in nightclubs as soon as he turned 18. In his twenties, he chose to go to the Helsinki casino to play, sometimes even in big poker tournaments, sometimes just for fun. When gambling at a casino, Mikko is fascinated by the sociability of gambling and the opportunity to utilize mathematical probabilities and gaming skills. Mikko has already lost his credit report for the second time and received several charges of fraud for taking short-term loans in the name of his father and his girlfriend.	(1) How and why has Mikko ended up in this situation? (2) What happens next? (3) Does Mikko need help in his situation? How could you help Mikko in social work?
Available service paths for the two case stories		(1) Are Mikko's and Aino's cases different from each other? How? (2) Are the available service paths suitable for them?

Following the discussions initiated by the casino gambling scenes, we presented our focus groups with the city's marketing video showcasing potential locations for the upcoming Tampere casino. This video acted as a bridge connecting casino gambling with the context of their work as municipal social workers in a city on the brink of experiencing a casino opening. Subsequently, the interviewer read two short stories about problematic gambling: the first concerning convenience gambling with EGMs in supermarkets and the second casino gambling. After discussing these stories, participants were asked about the possible service paths for both cases.

In the initial phase of our analysis, we applied an inductive approach to coding the data. We identified a wide range of harms caused by gambling that were expressed in the group discussions. Nonetheless, the harms described could be divided into two broad categories: financial and social, addressing both the individual and wider (family) levels. As we delved deeper into financial and social harms, we observed their interconnections: harm was often initially disguised primarily as financial.

We then observed how these interwoven harms were recognized as “gambling-related” in daily social work interaction; for example, by accident or through input from concerned significant others. Subsequently, we based the second round of coding on social workers’ responses to this “recognition of gambling harm”. These responses were labeled as barriers, suggestions and available services, further classified either as structural or individual issues. We also had an interest in the increase and diversification of gambling opportunities, with a focus on the Tampere casino. To this end, we also marked any mentions related to gambling availability. This allowed us to evaluate how the municipal social workers were prepared for the increase in local gambling opportunities.

Through collaborative discussions, we achieved a deeper understanding of the meaning-making logic within the data. We formed three thematic bundles within the group discussions, closely intertwined with social work and exhibiting significant overlaps. In the following sections, we present the participants’ unraveling of gambling-related harm in daily social work, along with their perceptions of existing structural and individual barriers in providing help for gambling-related harms.

## Results

### Gambling-related harm in daily encounters

The fictional gambling scenes and case stories served as stimuli for associations to “real-life” events and social work practice. The groups expressed that they often encountered gambling-related harm in their work: However, social work clients would seldom bring up gambling directly, even if it had already caused them serious harm. The study participants described typical cases of gambling-related harm as being discovered “by accident”. All groups highlighted that the most evident harms were financial difficulties, while their root cause (i.e. gambling) was often unclear and not so evident:

“[…] young people. Just the fact that even if things are completely messed up, the hair is great, and the clothes are neat. […] What's wrong with some services is that we think that the young man is doing really well, because his appearance is perfectly fine. But then, those things might be completely messed up, so maybe that type of young people get off our radar.” (Focus group 3)

In the excerpt above, a participant describes how a well-groomed appearance makes it challenging for professionals in welfare services detect behavioral dependencies compared to substance-related issues even when the client's financial hardship is obvious. In addition to detecting gambling-related issues, the groups also mentioned typical circumstances that could give rise to problematic gambling habits such as recent retirement: “before retirement, there should be some kind of screening for gambling in occupational healthcare, because it [gambling] is a big risk factor in that age group” (Focus group 4). According to the participants, it was quite common that concerned individuals, such as family members, contacted social services regarding older people: “The relatives have noticed and are worried about the elderly's potential gambling problem” (Focus group 4) and hence accidentally “reveal” someone else's gambling.

The groups unanimously agreed that financial harm was not purely economic; it was entangled with multiple issues such as family relations, secrecy, and financial abuse. The interviewees had discovered involuntary financial entanglements, such as grandchildren “robbing their grandparents” (Focus group 4) when scrutinizing their clients’ bank statements. Addressing sudden spending or money transfers with the client could lead to financial abuse unraveling or at times, a client would mention that the money goes to a grandchild's gambling habit. In many cases, the victims of financial abuse were unaware that they had been taken advantage of due to problem gambling: they believed that the money was needed for other purposes, such as “sending money back home” (Focus group 2). Sometimes, a close-one's gambling was discovered during an appointment:

“The wife comes [to an appointment] and says that there is not enough money in the family. […] And there is often a point where the wife is a client, and the husband's bank statement shows [money transfers to] gambling [companies] without the wife being able to say anything about it. […] I have to tell wives about their husbands’ gambling”. (Focus group 5)

”We have those couples where the husband has multiple payday loans and his credit's gone. But the wife is completely unaware: they know about financial problems or that the credit's gone, but they don’t necessarily know why. I mean, it's probably the case when you lose money, whether it's in a casino or payday loans or whatever, it's terribly embarrassing when you thought that you were going to save your family. And then, when it gets worse, it's such an embarrassing thing. You hardly dare say it right away. And then there can be aggression and everything when you try to hide it.” (Focus group 1)

The excerpt above illustrates the severity of shame related to problem gambling: “getting caught” can be so embarrassing that it leads to aggressive behavior. Despite the profound consequences, the focus groups noted that problem gambling was often considered secondary to substance-related dependencies which was also apparent in their daily work: “I think it's difficult to guide seniors anywhere just for their gambling addiction” (Focus group 2). In the conversation below, a group discusses how primary problems could, in fact, result from the problem being considered secondary:

**Participant 1:** “I feel that if there's also a substance use problem or a mental health problem besides the gambling problem, the focus is on those other problems and not on the gambling problem”.
**Participant 2:** “Maybe. […] A gambling problem is probably experienced, in social services and everywhere, as secondary in some way. It's not seen as big of a problem as it is. If there's alcohol or drugs involved, that's the primary concern. Right?”
**Participant 3:** “Sometimes things are so out of control that the most urgent thing is to secure housing, livelihood, and food. Clients’ situations are so problematic that other things come first.”
**Participant 1:** “Yes, and you don’t necessarily think that it could be the gambling addiction that has led to all these other problems.”
**Participant 2:** “And it probably has. As a result, people feel depressed and begin to drink or use drugs.” (Focus group 2)

The groups concluded that issues seemingly unrelated at first glance may have a strong association with problem gambling; however, the more visible or obvious problems often distract attention from recognizing the underlying issue.

### Barriers to help provision: lack of time and suitable services

Due to the wide range of clients’ life situations, the study participants viewed the existing service paths for gambling-related problems as insufficient. The main structural obstacles to the proper handling of clients’ gambling-related issues were, first, the social workers’ limited resources to address gambling properly even if they wished to help:

“Every time we meet a client, there are probably five to ten things already to address. If there is a need to treat this gambling issue, there's no way the working hours or appointments are enough. In fact, one's own professionalism is also sometimes lacking. How we can intensively help with that is the problem.” (Focus group 3)

“Sometimes it feels like everything's just exploding all the time and everything's chaotic with the clients. I'd like to support the clients.” (Focus group 3)

In addition to recognizing clients’ needs for support, but lacking time to address clients’ gambling issues during appointments, the second structural obstacle identified was the lack of suitable services for referring individual clients. The participants perceived the city's services as fragmented, noting that it did not provide a specialized “one-stop-shop” service for individuals facing gambling-related problems:

“If there was such a unit in Tampere, which is specialized specifically in addiction diseases, whether it was a gambling problem or some other functional disorder, drug addiction, whatever […] where addiction diseases are specifically understood, what it means to people, how to treat it.” (Focus group 3)

In the excerpt, a participant describes the need for integrated services in one location, rather than separate services for financial difficulties, health issues and social services.

The groups unanimously agreed that, in practice, they would recommend online therapy for all their clients even if they felt that the clients’ issues with gambling were more complex. However, they also noted that referral to online therapy was not always sufficient or suitable for their clients:

“Imagine if you told an alcoholic to just go home and not drink and read some stuff on the internet. Anyway, when it comes to treating gambling problems, it's not in good hands.” (Focus group 5)

By comparing gambling to other dependencies, a participant highlights the attitudes towards gambling problems: it would be inappropriate to send an individual with an alcohol problem home to simply read something on the internet, whereas this is typical with gambling problems. The participants also noted the contradictory role of the City of Tampere in applying to host a casino. They viewed the casino as conflicting with the city's aims of reducing gambling-related harm and its responsibilities toward residents:

“If Tampere is really trying to reduce gambling problems. Well then, there are certainly many players out there who don’t have a gambling problem yet. […] I understand that private companies are interested [in having casinos] but the city.” (Focus group 5)

In other words, a participant suggests that if the city truly wanted to reduce gambling harm, it should establish proper treatment services first, before applying to host a casino, knowing that it would inevitably lead to gambling-related harm.

Another participant emphasized the need for diverse services due to the varying needs of individuals:

“Our clients wouldn’t go near any groups [for problem gambling] […] the shame of a gambling problem is way too much. They would never show their face there.” (Focus group 3)

The participant explains that shame is a significant barrier preventing some clients from attending peer support groups. However, they also note that this does not mean that a client rejects treatment altogether. Online anonymous groups and individual therapy are much-needed options**.** One group also brought up the range of individuals in inpatient treatment: “Even though substance use treatment centers also treat people with gambling problems, in my opinion, that can be a bit too much [for individuals with gambling-related problems]” (Focus group 1). Therefore, they also recommended specialized groups for gambling issues in treatment, rather than viewing all dependencies as the same.

Despite their feelings of insufficient or unsuitable services for their clients, our participants still emphasized the importance of inquiring about clients’ gambling. They discussed how the early identification of gambling problems was the most effective step in reducing gambling harms and highlighted that addressing clients’ problems was at the core of their work:

“We do dare to ask about domestic violence, gambling problems, and everything. Clients trust us with their bank statements and listen to us, but when it comes to the next steps, referral to further treatment is a problem.” (Focus group 3)

While the willingness to address gambling was evident, the social workers’ primary concern was the lack of available and suitable treatment opportunities, along with their limited time to thoroughly address the issue during appointments.

### Barriers to help-seeking: stigma, shame and false conceptions

Besides structural barriers, our groups viewed that many clients had subjective barriers to help-seeking as well as false conceptions of gambling. They described the feelings caused by problem gambling in strong words such as “shame” and “despair”. The emotional intensity of shame was discussed extensively, with participants noting that it can be so overwhelming that it prevents individuals from even mentioning gambling and thus from seeking help. Our participants evaluated that it was rare, and considered it brave, for clients to speak about their problems related to addictive substances or behavior:

“Rarely, when a person openly talks about their substance use problem, we easily just list things that they need to do. Bringing it up should lead to collaborative thinking without blaming and moralizing. Through that, stigma could diminish. Maybe the willingness to receive help could arise then. It shouldn’t cause any kind of shame. That's why it would be important to address this [gambling] with everyone [every client], not just some. Another question is what to do next.” (Focus group 4)

In the preceding excerpt, a participant emphasizes the importance of refraining from moralizing or lecturing clients. The participant recognizes the potential of social work in reducing stigma by incorporating a standard questionnaire about gambling for every client. This would normalize discussions about gambling-related harm to the clients as well.

Our groups paid attention to understanding clients’ gambling motivations and subjective barriers to help-seeking: these motivations stemmed from shame and despair, which intertwined with clients’ attempts to win back the losses. The groups viewed typical misconceptions of winning and earning money by gambling as key factors preventing clients from help-seeking:

**Participant 1:** “A desperate act. I’ve met some [clients] who have debt and a gambling problem. Many times, they say that somehow, they feel like they have to gamble. […] They feel that it's the only way to solve the problem; that, ‘now I have to gamble and take a loan. If I won, I could pay off all these debts.’”
**Participant 2:** “I’ve also come across clients who say that they don’t get enough money. More money must be gotten from somewhere. And gambling is the only way; they don’t consider getting a job as an option, gambling's a quick fix. […] I feel that they are no longer comfortable gambling. It's obsessive because of their despair.” (Focus group 5)

Two groups mentioned that the casino could potentially generate false conceptions about gambling: Some clients may consider casinos not as harmful due to their image of affluent middle-class casino clientele. It might foster the belief that gambling is a profitable activity:

“[If] people with gambling problems and in need of social work go there and see rich people who gamble. The delusion that you can actually win by gambling is probably even stronger. Although it's quite likely that those rich-looking people got their money from somewhere else than the casino.” (Focus group 5)

The groups highlighted the role of empathy when addressing gambling issues with clients. They acknowledged that false perceptions should be taken seriously because clients may genuinely believe in them and view gambling as “a way out of misery” (Focus Group 1), which can further deepen the harms experienced. They also emphasized that addressing clients’ gambling should be a standard procedure: that way the clients “wouldn’t feel that they’re the only one being interrogated” (Focus group 2). This approach could also reduce self-stigmatization.

## Discussion

The present study has investigated how gambling-related harm intersects with municipal social work encounters and how social workers perceive the available service pathways on the verge of the introduction of new local gambling opportunities. It documents municipal social workers perceptions before the opening of a local casino and before a major health and social services reform in the Finnish system. Employing a qualitative approach, five focus group interviews were conducted involving 15 social workers. The findings elucidate the range of harms arising from gambling, often unintentionally unraveled in daily social work encounters. The focus groups acknowledged the existing impediments to help-seeking behavior due to stigma, shame and misconceptions about gambling and winning, alongside barriers in the availability of services for problem gambling. However, while participants acknowledged a connection between gambling availability and harm, they did not view the casino as the type of gambling opportunity that would appeal to their clients. Similarly, the broader audience appeared uninterested because the casino closed just 2 years after its opening (for the Tampere residents’ perceptions, see Jääskeläinen et al., 2021).

While our study shows that municipal social workers encounter gambling-related harm in their daily work and are eager to help, the time and available services are insufficient to meet the diverse needs of all clients. Furthermore, there is still no standard protocol for addressing gambling-related questions in social work, even though guides and questionnaires on the issue are available for professionals (Björkenheim et al., 2021; Järvinen-Tassopoulos & Kesänen, 2021). In 2023, the first national independent, evidence-based clinical practice guidelines (Current Care Guidelines) for problem gambling were finally introduced.

Given the prevalence and the normalized status of gambling in Finland, this discrepancy is concerning. Problem gambling is often addressed (also in social work) as secondary to other urgent needs, receiving less attention than other dependencies (Nower et al., 2023; Rogers, 2013). This need can be demonstrated through Finnish inpatient care, in which group therapy is commonly organized within a broader framework of addiction treatment, encompassing substance and behavioral dependencies alike (Nevalainen et al., 2022). Nevertheless, there is no available information on whether individuals with gambling problems benefit from such general groups that mainly consist of individuals with substance use problems.

Therefore, future studies should address this “secondary problem” dimension of addressing gambling issues in social work. Scientific knowledge on both the clients’ treatment needs and the types of gambling harms, including shame, stigma, and cultural barriers to help-seeking, is needed to enhance proper referral to diverse services for different ages, genders and ethnic groups, as well as for diverse forms of gambling ranging from “luxury” casinos to online gambling that no one sees.

This study has some limitations. Notably, despite involving multiple participants in focus group interviews, inherent sampling bias may affect the generalizability of the findings**.** The subjective views expressed by participants might not fully represent the diversity of perspectives within the field of municipal social work as services vary depending on the municipality and wellbeing services county. Furthermore, using focus groups as a data collection method introduces the risk of social desirability bias, particularly because participants were encouraged by their supervisor to take part. Additionally, online focus group interviews may lack the spontaneity of in-person interviews.

Finally, our study highlights the importance of screening for problem gambling in social work: it underscores the urgent need for a clear strategy for addressing gambling issues, including early interventions and diverse, accessible service provision. Normalizing gambling-related discussions within social work settings could reduce stigma and enhance knowledge and empathy among practitioners (Gainsbury et al., 2014). Including clients’ gambling behavior on the agenda of social work would also benefit treatment provision, given the higher prevalence of gambling problems among individuals with substance use and mental health issues (Castrén et al., 2013; Heiskanen & Kuussaari, 2023; Lorains et al., 2011). We recommend that policymakers establish centralized, comprehensive services for gambling problems to support individuals grappling with gambling-related issues.
